# Natural history and morphology of the hoverfly *Pseudomicrodon biluminiferus* and its parasitic relationship with ants nesting in bromeliads

**DOI:** 10.1093/jis/14.1.38

**Published:** 2014-01-01

**Authors:** Volker S. Schmid, Mírian N. Morales, Luciane Marinoni, Rafael Kamke, Josefina Steiner, Anne Zillikens

**Affiliations:** 1 Biologie I, Universität Regensburg, 93040 Regensburg, Germany; 2 Med.–Naturwissenschaftliches Forschungszentrum, Ob dem Himmelreich 7, Universität Tübingen, 72074 Tübingen, Germany; 3 Universidade Federal do Paraná – UFPR, Dept. de Zoologia, Pós-graduação em Entomologia, Cx. Postal 19020, 81531-980 Curitiba, Paraná, Brazil; 4 Universidade Federal de Santa Catarina, Laboratório de Abelhas Nativas (LANUFSC), Centro de Ciências Biológicas, Campus Universitário Trindade, 88040-900 Florianópolis, Santa Catarina, Brazil

**Keywords:** Brazil, *Camponotus*, *Crematogaster*, Formicidae, host record, Microdontinae, myrmecophily, Neotropics, Syrphidae

## Abstract

The syrphid subfamily Microdontinae is characterized by myrmecophily of their immature stages, i.e., they develop in ant nests. Data on natural history of microdontines are scarce, especially in the Neotropics. Based on fieldwork in southern Brazil, this study provided new data on development and ecology of the hoverfly *Pseudomicrodon biluminiferus* (Hull) (Diptera: Syrphidae) as well as the first morphological descriptions of male genitalia, larvae, and pupa. Immature specimens were specifically found in colonies of the ant species *Crematogaster limata* Smith (Hymenoptera: Formicidae) found in rosettes of the bromeliad species *Aechmea lindenii* (E. Morren) Baker (Poales: Bromeliaceae) and *A. nudicaulis* (L.) Grisebach. Third instar larvae were observed preying on ant larvae, revealing the parasitic nature of *P. biluminiferus* . In this and several other aspects, the natural history of *P. biluminiferus* is similar to that of Holarctic microdontine species. Exceptions include: (i) indications that adults of *P. biluminiferus* outlast the winter months (in contrast to 3 ^rd^ instar larvae in Holarctic species) and (ii) *P. biluminiferus* ’ relationship with bromeliads. The importance of bromeliads for this host-parasite system is evaluated in this paper. The single occurrence of another, unidentified microdontine species’ pupae in a nest of the ant species *Camponotus melanoticus* Emery (Hymenoptera: Formicidae) is reported.

## Introduction

Colonies of social insects like ants, bees, wasps, and termites provide a very beneficial environment for their brood that includes good nutrition, shelter, favorable climatic conditions, and protection against predators. A highly diverse range of species (called myrmecophiles if associated with ants) has evolved to utilize these benefits for their own development by living inside social insect colonies as either mutualists, commensals, or parasites ( Wilson 1971 ; Kistner 1982 ; Hölldobler and Wilson 1990 ; Lachaud et al. 2012 ). Among myrmecophilous species, those of the syrphid subfamily Microdontinae stand out due to the strange slug-or coccid-like shape and movements of their larvae that caused taxonomic confusion until the early 20th century ( Wheeler 1908 ). Moreover, 454 species of Microdontinae have been described worldwide ( Reemer 2013a ), but biology and host relationships have been studied for only a few, which are mostly Holarctic species.

All three larval stages of most microdontine species occur in nests of ants ( Wheeler 1908 ; Andries 1912 ; Akre et al. 1973 ; Duffield 1981 ). Field populations of host ants have been shown to be infested by microdontine brood at rates that range from 16% ( Akre et al. 1973 ) to 33–50% of nests ( van Pelt and van Pelt 1972 ). The reported number of microdontine brood items per ant nest varied greatly, from two ( van Pelt and van Pelt 1972 ) to more than 240 ( Akre et al. 1973 ), and reported means ranged from three to six brood items ( Duffield 1981 ; Schönrogge et al. 2002 ). Most microdontine species developed one brood generation per year ( Akre et al. 1988 ); the species *Microdon fuscipennis* Macquart (Diptera: Syrphidae) develops at least one generation yearly, and some species develop more than one ( Duffield 1981 ). In North American and European species, 3 ^rd^ instar larvae have been observed to overwinter in their hosts’ nests ( Andries 1912 ; Garnett et al. 1985 ; Akre et al. 1990 ).

Larvae of several microdontine species have been reported to feed on their hosts’ brood ( Hocking 1970 ; van Pelt and van Pelt 1972 ; Duffield 1981 ; Garnett et al. 1985 ; Barr 1995 ). In this context, the larvae can be regarded as either predators of the ants’ brood or parasites of an infested colony as a whole; as a “superorganism” ( Hölldobler and Wilson 2009 ), the colony may be subject to group-level selection. This is the manner in which the terms predation and parasitism will be used throughout this article. A distinct case is primary parasitism of Microdontinae as reported for the species *Hypselosyrphus trigonus* , which infests colonies of *Pachycondyla villosa* ( Pérez-Lachaud et al. 2014 ).

Pupation of microdontine larvae takes place near the surface, where entrances of the nests are found ( Wheeler 1908 ; Duffield 1981 ; Garnett et al. 1985 ; Akre et al. 1988 ; but see Andries 1912 ). The dorsal surface of the immature stages of development is convexly curved, a characteristic that is most pronounced in 3 ^rd^ instar larvae and pupae, and covered with a distinct pattern of tubercles, reticulations, or similar structures. The larvae display a posterodorsal stigmatic scar, and pupae are additionally characterized by two small anterodorsal stigmatic horns ( Andries 1912 ; Garnett et al. 1990 ).

The microdontine pupal stage lasts 11–28 days ( Andries 1912 ; Greene 1923b ; Jordan 1968 ; van Pelt and van Pelt 1972 ; Akre et al. 1973 ). Emergence of adults occurs in the early morning or at night. The mere process of emergence requires one to a few minutes, whereas wing expansion takes at least 5–10 minutes and up to several hours ( Wheeler 1908 ; Andries 1912 ; van Pelt and van Pelt 1972 ; Akre et al. 1973 , 1988; Duffield 1981 ; Forti et al. 2007 ). Sex ratios near 1:1 have been reported ( Akre et al. 1973 , 1988; Duffield 1981 ).

Microdontine brood has been reported to be associated with various ant species, predominantly of the genera *Formica* and *Camponotus* (subfamily Formicinae) ( Duffield and Thompson 1981 ; Reemer 2013a ). Microdontine larvae and pupae are usually treated indifferently by their hosts ( Wheeler 1908 , 1910; Jordan 1968 ; Akre et al. 1973 ; Garnett et al. 1985 , but see van Pelt and van Pelt 1972 , who reported that ants investigated and killed some microdontine larvae) or even transported like ant brood by worker ants ( Garnett et al. 1985 ). Similarly indifferent ant behavior toward microdontine imagines immediately after emergence was described by Wheeler (1908) . On the other hand, Andries (1912) and Akre et al. (1973) observed that adult microdontines were immediately attacked by ants upon emergence.

At present, few publications about microdontine-ant associations in South America are available, and in most cases the syrphid species was not identified, such as in Paraguay ( Sharp 1899 ), Guyana ( Wheeler 1924 ), and Brazil ( Borgmeier 1923 ; Luederwaldt 1926 ). Only Forti et al. (2007) reported a fully identified association in Brazil, in which *Microdon tigrinus* Curran (Diptera: Syrphidae) lived in nests of leaf-cutter ants, *Acromyrmex coronatus* (F.) (Hymenoptera: Formicidae). Reports from Central America are also scarce, and include the countries of Panama ( Wheeler 1924 ; Mann 1928 ) and Costa Rica ( Longino 2003a ). There are few studies of microdontine-ant myrmecophily in Africa ( Wasmann 1894 ; Speiser 1913 ; Hocking 1970 ), Australia ( Buschinger 1998 ), and Asia ( Hironaga and Maruyama 2004 ). A comprehensive overview of the worldwide distribution of reports on microdontine-ant associations was recently provided by Reemer (2013a) .

An important condition for the study of microdontine-ant relationships is the ability to identify larvae and pupae, because it is not always feasible to rear adults ( Garnett et al. 1990 ). Detailed knowledge of the morphology of immature developmental stages might also be useful for analyzing the phylogeny of Microdontinae. While descriptions and identification keys to immature stages are available for some Holarctic species ( Wheeler 1908 , 1910; Greene 1923a , 1923b, 1955; Dixon 1960; Novak et al. 1977 ; Garnett et al. 1990 ), no keys and only one general description for a Neotropical species ( Wheeler 1924 ) were available.

During fieldwork in secondary forests of coastal southern Brazil, microdontine pupae were found in bromeliads (Bromeliaceae) inhabited by ants. Bromeliaceae are a monocot plant family mainly distributed in the Neotropics and neighboring subtropical regions ( Benzing 2000 ). Within the microcosms of their leaf rosettes, they harbor a highly diverse range of aquatic and terrestrial animals that are frequently associated with ants ( Dejean and Olmsted 1997 ; Frank and Lounibos 2008 ; Camargo and Oliveira 2012 ). In spite of this diversity, no immature specimens of microdontine species had been found or identified in bromeliads prior to the current study.

In order to obtain more data about immature stages of the hoverfly *Pseudomicrodon biluminiferus* (Hull) (Diptera: Syrphidae) and to analyze the animal-bromeliad relationship, there were two aims of the present study. The first was to describe the biology, ecology, and host-parasite interactions of the Neotropical species *P. biluminiferus* for comparison with species of temperate regions. The second aim was to provide detailed morphological descriptions of immature stages and adults. During the authors’ research on *P. biluminiferus* , immature specimens of another microdontine species were found. Data on this finding are presented and briefly discussed. Aside from morphology, most results about microdontine larvae concern the 3 ^rd^ instar because 1 ^st^ and 2 ^nd^ instar larvae were rarely found.

## Materials and Methods

### Sample acquisition and studies on developmental biology and ecology

**Figure 1.**
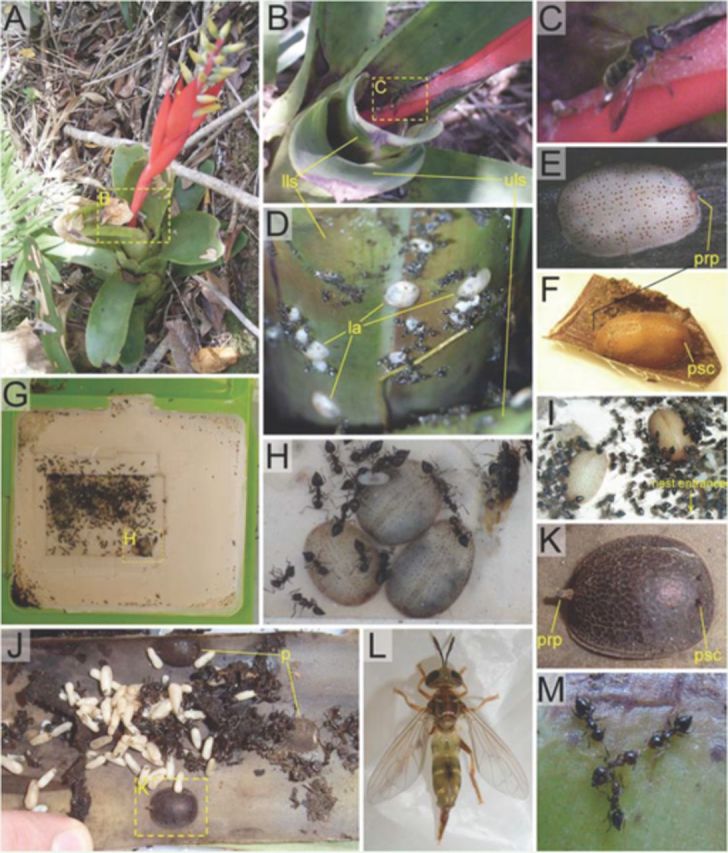
Photo plate. Dashed frames denote a more detailed illustration of the same structure/context in another picture; only C is a pure digital enlargement of the source picture (B), whereas all other cases originate from distinct photos. “la” refers to microdontine larvae, “p” to puparia, “lls” to lower leaf surface, “uls” to upper leaf surface, “prp” to posterior respiratory process, and “psc” to pupal spiracle. **A.** Flowering bromeliad ( *Aechmea nudicaulis* )*. **B.** Central bromeliad rosette ( *A. nudicaulis* ) with base of inflorescence stem. **C.** Syrphid at stem base of bromeliad inflorescence ( *A. nudicaulis* ). **D.** Ant nest ( *Crematogaster limata* ) containing *Pseudomicrodon biluminiferus* larvae (la) within bromeliad rosette ( *Aechmea* sp.). **E.** Larva of *P. biluminiferus* found within ant nest ( *C. limata* ) in stem base of withered bromeliad inflorescence ( *Vriesea friburgensis* ). **F.** Pupa of *P. biluminiferus* found in ant nest ( *C. limata* ) within bromeliad rosette ( *Aechmea* sp.). **G.** Laboratory nest (depression in plaster, covered with glass plates) with ant colony ( *C. limata* ); top: opening to foraging arena. **H.** Third-instar larvae of *P. biluminiferus* (same individuals as in G) in laboratory ant nest. **I.** Young pupa (right) and mature larva or prepupa (left) of *P. biluminiferus* in laboratory ant nest ( *C. limata* ); the broad nest entrance is located below the lower edge of the picture. **J.** Bromeliad rosette leaf ( *A. nudicaulis* ) harboring ant colony ( *Camponotus melanoticus* ) with one pupa (dashed frame) and two puparia (p) of Microdontinae sp. 1; note anterior ends of all three pupae/puparia directed toward leaf tip (beyond right edge of picture). **K.** Pupa of Microdontinae sp. 1 (same as in J). **L.** Teneral *P. biluminiferus* female extending wings and ovipositor after emergence. **M.** Worker ant ( *C. limata* , middle) transferred into near conspecific field nest and attacked by three surrounding conspecifics. *For pictures of the related bromeliad species *A. lindenii* , in whose rosettes microdontine brood were also found, see Schmid et al. 2010 . High quality figures are available online.

Fieldwork was carried out between December 2005 and April 2010 on a mountainside covered with secondary forest in Santo Antônio de Lisboa (27° 30’ S, 48° 30’ W) within the district of Florianópolis on Santa Catarina Island in southern Brazil. The first microdontine specimens were found in ant nests within terrestrial bromeliad rosettes ( Figure 1A , B , D ). To find more specimens, about 300–400 bromeliad rosettes were examined in the field along trails from August to February, and once during April. Bromeliad rosettes were mainly of the species *Aechmea lindenii* (E. Morren) Baker (Poales: Bromeliaceae) ( Schmid et al. 2010 ) and *A. nudicaulis* (L.) Grisebach ( Figure 1 A, B), and were examined for the presence of ants and immature microdontine specimens ( Figure 1D , E ). To assess the infestation rate of ant colonies by Microdontinae, 33 ant nests within *Aechmea* spp. were examined for microdontine brood on 5 November ( *N* = 20), 15 November ( *N* = 8), and 4 December 2008 ( *N* = 5), in a different section of the forest each time. To estimate the number of brood items per colony, five ant nests were thoroughly searched for microdontine larvae, pupae, and fresh puparia by breaking the bromeliad rosettes apart leaf by leaf.

To obtain adults, three bromeliads containing ant colonies with *P. biluminiferus* brood were taken into the laboratory, and each was placed into a bucket and enclosed with a fine gauze net. Every day the nets were checked for adult flies. Twenty pupae and one 3 ^rd^ instar larva collected from bromeliads in the field or in the laboratory were individually placed in transparent vials with a piece of wet paper to provide humidity.

**Figure 5.**
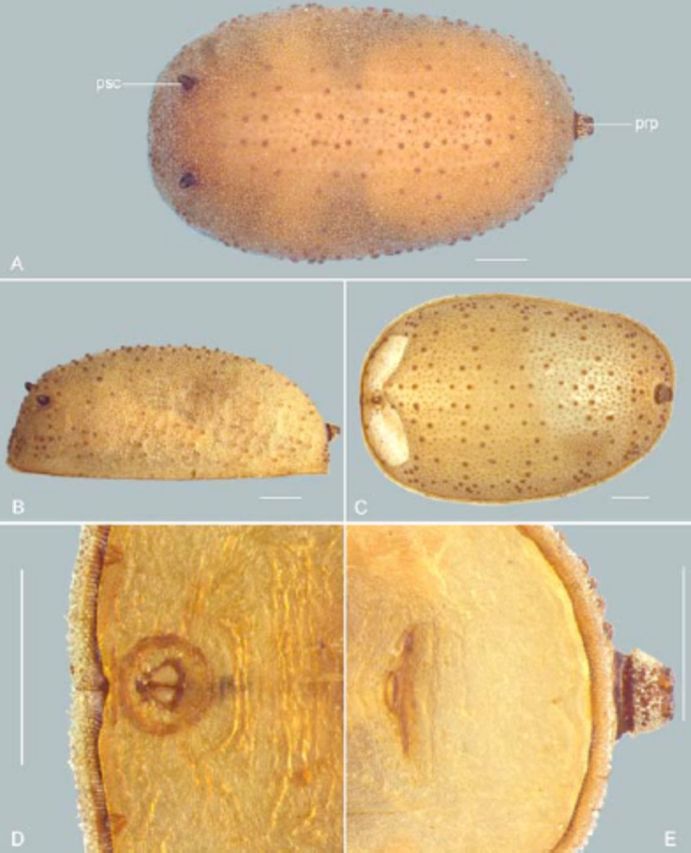
*Pseudomicrodon biluminiferus* . *Pupa:***A.** Dorsal view. **B.** Lateral view. *Puparium:***C.** Dorsal view. *Pupa, ventral view:***D.** Detail of oral scar. **E.** Detail of anal scar. Scale bar: 1 mm; “prp” refers to posterior respiratory process, and “psc” to pupal spiracle. High quality figures are available online.

A distinct change of the larval body toward the slightly higher and more slender pupal shape ( Figure 1 F, I ) was interpreted as pupation, marking the beginning of the pupal stage; a similar change has been described in other microdontine species ( Wheeler 1908 ; Andries 1912 ). This definition might include the prepupal phase ( van Pelt and van Pelt 1972 ), since other authors have defined pupation as beginning “only when the anterior spiracles appeared” ( Akre et al. 1973 ) (“psc” in Figures 1 , 5 ). Because the time of appearance of those spiracles was not noted in our study, only the first pupal stage definition given above could be applied in the current study, which is perhaps an overestimation of the true pupal stage by 1–3 days. Twenty-six adults were obtained altogether, 19 of which could be associated either with an exact emergence time ( *N* = 4), a reasonable estimate of emergence time (e.g., discovery before wing expansion; *N* = 3), or the time of first sighting ( *N* = 12). Exact emergence time was obtained by serial photography at a rate of one picture every 10 seconds using a Caplio R5 digital camera (Ricoh, www.ricoh.com ), or by video with a Handycam HDR-SR10E camcorder (Sony, www.sony.com ).

The identity of *P. biluminiferus* , which was initially named *Microdon biluminiferus* , was confirmed by F. Christian Thompson, who compared adult specimens with the holotype deposited in the Naturhistorisches Museum Wien, Vienna, Austria. Ants were identified by comparison with samples in the collection of the Native Bee Laboratory of the Federal

University of Santa Catarina, Florianópolis, Brazil. Time specification is given in 24-hour clock notation. Sex ratio was statistically compared to a hypothetical 1:1 ratio by performing a Chisquare test using a custom-made Excel workbook (CHISQ 1.0.0 by Peter Pilz, University of Tübingen, Germany, 2005) (Microsoft, www.microsoft.com ).

### Behavioral studies

To observe the behavior of *P. biluminiferus* larvae and ants toward one another, two artificial laboratory nests consisting of a depression in plaster covered with glass plates were constructed ( Figure 1G ). Laboratory nests and vials were stored in rooms without climatic and light period control, but were always close to windows so that light conditions were presumably similar to moderately open forest.

First, one colony of the ant host species, *Crematogaster limata* Smith (Hymenoptera: Formicidae), was transferred into an artificial nest ( Figure 1G , H , I ). The colony contained about 200–300 workers, several winged sexu-als, a few dozen ant brood items of different developmental stages, and three 3 ^rd^ instar *Pseudomicrodon* larvae. To examine whether *Pseudomicrodon* larvae are specifically integrated into their host nests or whether they are generally ignored by their hosts, two 3 ^rd^ instar *Pseudomicrodon* larvae from another *C. limata* colony were placed into the first nest (as described above) after the three initially-present *Pseudomicrodon* larvae had pupated. To obtain an additional (though not completely independent) replicate, the two transferred larvae were moved two weeks later into the second artificial nest, which contained yet another *C. limata* colony. Behavior of ants and *Pseudomicrodon* larvae was observed for approximately 3–5 minutes immediately after transfer as well as occasionally during the following two weeks. Larval behavior was recorded with a Handycam HDR-SR10E camcorder (Sony) five times prior to manipulation and twice post-transfer; total duration of video recording was 37 min. Ant and *Pseudomicrodon* larvae behavior was also examined for ant-syrphid interactions, as was the emergence of one adult in a laboratory ant nest.

To test whether lack of aggression by host ants is a general trait of colonies in the study area, a field experiment was conducted. From each of two bromeliad-inhabiting *Cr. limata* colonies (donors, both containing *Pseudomicrodon* larvae), five worker ants were transferred into two other ant nests (receivers, one with and one without *Pseudomicrodon* larvae). Donor colonies were 3 m apart from receiver colonies and situated in different groups of rosettes than the receiver colonies. In addition, two *Pseudomicrodon* larvae were moved between nests during this aggression test. The authors observed whether the transferred ants were treated aggressively by the host ants in each nest.

### Morphological studies

Larvae were fixed in Kahle’s solution and preserved in 70% ethanol. Voucher specimens of microdontine larvae and adults, as well as associated ants, were deposited in the collection of the Native Bee Laboratory of the Federal University of Santa Catarina and Father Jesus S. Moure Entomological Collection, Department of Zoology, Federal University of Paraná, Curitiba. The morphology of the immature specimens and adults was examined with an MZ 75 stereomicroscope (Leica, www.leica.com ). Male genitalia were cleared in a 10% KOH solution for 36 hours, neutralized with glacial acetic acid, washed with 70% ethanol and then distilled water, and stored in glycerol.

Light micrographs were obtained with a DFC 500 digital camera attached to an MZ 16 stereomicroscope (Leica). Images were captured using IM 50 software (Leica), then mounted using Automontage software (Syncroscopy, www.syncroscopy.com ). Scanning electron microscopy images were obtained with a JSM-6360 LV microscope (JEOL, www.jeol.com ) in order to study details of the 3 ^rd^ instar larval cuticle, posterior respiratory process, marginal band, ventral surface, and pupal spiracle. Terminology is derived from Roberts (1970) and Rotheray and Gilbert (1999) for larvae, and from Thompson (1999) for adults.

## Results

### Discovery of two Microdontinae species

Immature specimens of two Microdontinae species were found within ant colonies in bromeliad rosettes ( Figure 1 D– F , J , K ) on Santa Catarina Island in southern Brazil. At least 30 larvae and 21 pupae of *P. biluminiferus* were collected, 26 of which were reared to adults. Two puparia and one pupa of a second species of Microdontinae (Microdontinae sp. 1) were found in a queenless nest of *Camponotus melanoticus* Emery (Hymenoptera: Formicidae) within a rosette of *A. nudicaulis* , but could not be reared to imago. The same rosette was also inhabited by a colony of *Crematogaster limata* infested with *P. biluminiferus* . Pupae of the two microdontine species could be readily distinguished ( Figure 1F , K ).

### Biology of *Pseudomicrodon biluminiferus*

Both in the field and in the laboratory, pupae and puparia were frequently located near the nest entrance and/or with their anterior ends directed toward the opening ( Figure 1 I, Videos 1 , 2 ), while larvae were additionally scattered throughout other nest parts ( Figure 1D ). When dates were compiled across all years (comprising April and the months from August to February), larvae were found between early November and mid-December as well as on 16 April 2010. From the field colonies taken to the laboratory, seven *Pseudomicrodon* imagines emerged. Out of the 21 immature specimens placed in vials, 16 imagines were obtained. Two failed to expand their wings, which appears to be a laboratory effect that has been reported elsewhere (Jordan 1968; Akre et al. 1973 , 1988). Three individuals pupated and emerged within laboratory ant nests. For those three specimens and one larva that pupated in a vial, the duration of the pupal stage was determined to last 18 days ( *N* = 1) or 19 days ( *N* = 3).

**Video 1.** *Pseudomicrodon biluminiferus* adult emerging from pupa (the middle one) within laboratory ant nest ( *Crematogaster limata* ). Available at www.insectscience.org/14.38/Schmidvid1.avi

**Video 2.** *Pseudomicrodon biluminiferus* adult emerging from pupa on natural substrate (bromeliad leaf). Note that the leaf tip is located beyond the left edge of the video frame where remains of a carton-like wall (presumably constructed by *C. limata* ants) can be seen. Available at www.insectscience.org/14.38/Schmidvid2.avi

**Figure 2.**
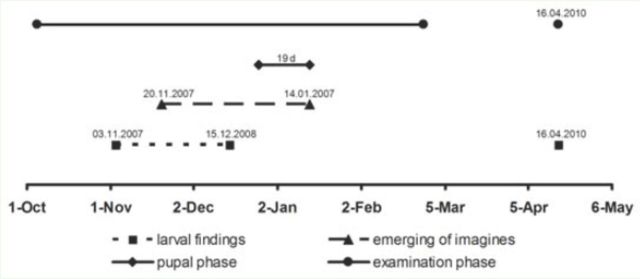
Larval and adult seasons estimated from searches in the field (August to February and April) and emergences in the laboratory. The median pupal phase (19 days; *N* = 4) was arbitrarily placed at the end of the recorded emerging phase for comparison. Dates used for compiling the phases are given above the corresponding points. High quality figures are available online.

Imagines emerged between 20 November and mid-January ( Figure 2 ). Live pupae were not found outside of this period. Occasional searches for microdontine brood in *Crematogaster* nests in the field from August to October as well as late January and February resulted in no findings. Thirteen of 19 adults (68%) emerged before 08:00. Most remaining individuals were found later in the day (09:40 to 12:38), but since they were no longer in wing expansion posture, they might also have emerged in the early morning. One exception was an individual found at 17:00, whose pupa had been noted as being closed at 10:00. For seven individuals, the exact time of emergence could be determined or estimated as being between 05:57 and 08:25.

Emergence took between 0.5 and 1.5 min from the first appearance of the head to the first steps of the adult outside the puparium ( Videos 1 , 2 ). First signs of imminent eclosion were visible starting approximately 20 min before actual emergence ( Video 2 ) and consisted of the following: (1) a darkening of the anterior end of the pupa, probably due to the inner surface getting wet, (2) the three anterior plates (surrounded by lines of weakness) being pushed outward several times without the head emerging clearly, and (3) movements of the adult within the puparium. The single adult that was observed eclosing within an ant nest walked straight toward the nest entrance and quickly left the nest ( Video 1 ). After emergence, adults moved to an elevated place (a wet paper towel or a bromeliad leaf) and expanded their wings within about half an hour ( Figure 1 L, Video 3 ). Sex ratio was 8:5 (female:male) and did not differ significantly from a 1:1 distribution ( *χ ^2^* = .69, *p* = .4054, *Ntotal* = 13).

**Video 3.** *Pseudomicrodon biluminiferus* adult expanding its wings and extending its proboscis in the laboratory. Available online at www.insectscience.org/14.38/Schmidvid3.avi

### Descriptions of *Pseudomicrodon biluminiferus*

**Figure 3.**
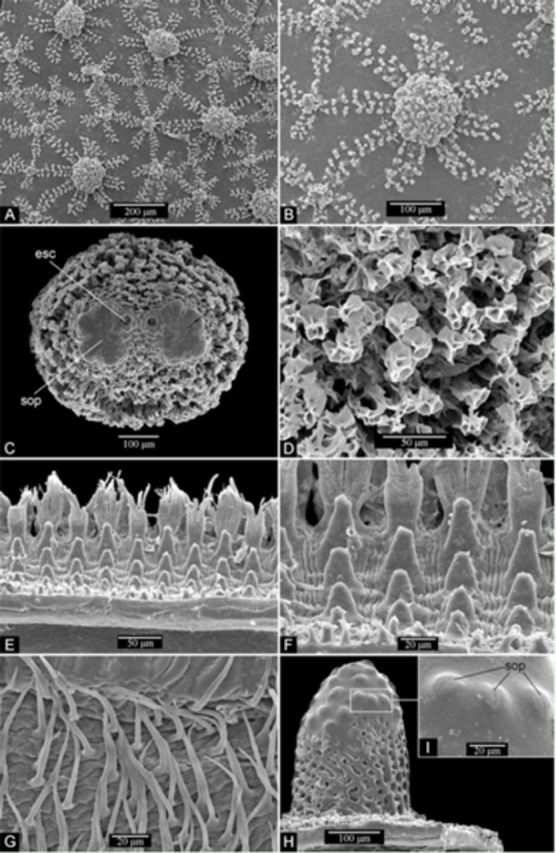
*Pseudomicrodon biluminiferus* . Scanning electron microscopy images. *3rd instar larva:***A.** Reticulate pattern on tegument. **B.** Detail of a big wart-like process on tegument. **C.** Posterior respiratory process, dorsal view. **D.** Posterior respiratory process, detail of lateral surface. **E.** Marginal band, dorsal view. **F.** Marginal band, detail of papilliform protuberances. **G.** Ventral surface, detail of ventrolateral pubescence. *Pupa:***H.** Pupal spiracle, lateral view. **I.** Detail of spiracular openings. “sop” refers to spiracular opening, “esc” to ecdysial scar. High quality figures are available online.

**Figure 4.**
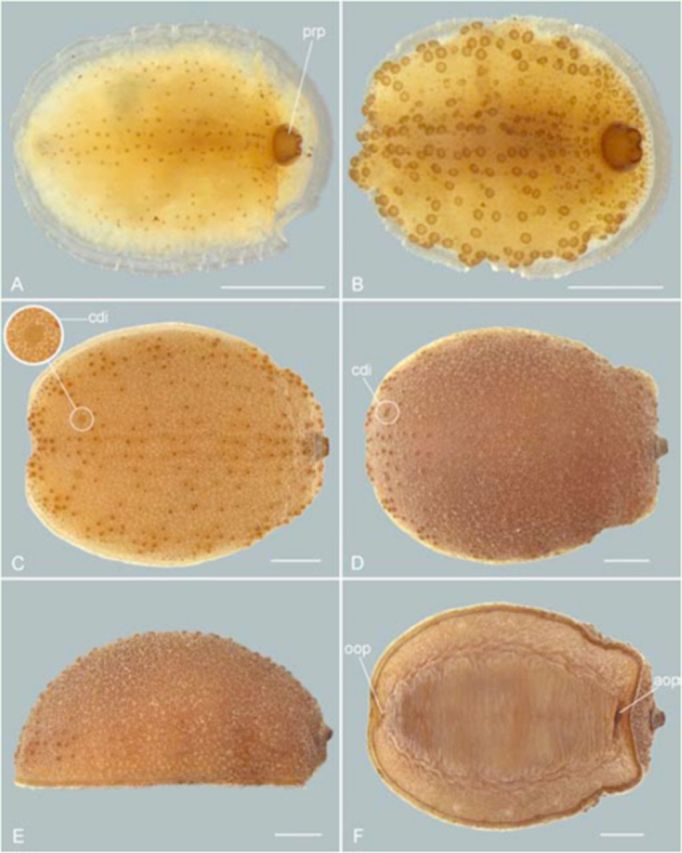
*Pseudomicrodon biluminiferus* . **A** . 1st instar larva, dorsal view. **B.** 2nd instar larva, dorsal view. **C.** 3rd instar larva, dorsal view. *Prepupa:***D.** Dorsal view. **E.** Lateral view. **F.** Ventral view. Scale bar: 1 mm; “aop” refers to anal opening, “cdi” means slightly convex disc without reticulation, “oop” refers to oral opening, and “prp” to posterior respiratory process. High quality figures are available online.

Third instar larva. ( Figures 3A–G , 4C ). Length: 6.06–6.84 mm, maximum width: 5.25–5.27 mm ( *N* = 2). Hemispherical, buff in color. Cephalic segment retracted, mouthparts reduced and internal (as shown for prepupa, Figure 4 F). Dorsally from metathorax to posterior end strongly convex, with a reticulate pattern formed by line arrangements of granulation radiating from numerous wart-like processes of different sizes ( Figure 3A , B ). Bigger processes with eight to nine rays, smaller ones with four to six. Posterior respiratory process sessile, trapezoidal in anterior and posterior view ( Figures 3C , 4C ), circular basally (dorsal view). Apex of posterior respiratory process constituted by four flat emarginations, ecdysial scars oval ( Figure 3 C), spiracular openings indistinct ( Figure 3 C), cuticle rough ( Figure 3 D). Ventral surface flattened, ventrolateral pubescence consisting of fine setae ( Figure 3 G). Prolegs and crochets absent. Marginal band ( Figure 3 E, F): distal portion with two rows of multi-branched flattened setae, proximal portion with three to four lines of papilliform protuberances.

Instar identification. *Pseudomicrodon biluminiferus* has three larval instars, which can be distinguished by differences in dorsal reticulation process patterns and developmental degree of the posterior respiratory process. In the first instar, dorsal reticulation processes are scant, inconspicuous, and composed of little brown rounded shapes ( Figure 4 A); apex of posterior respiratory process is not differentiated in emarginations. In the 2 ^nd^ instar, dorsal reticulation processes are larger and partially sclerotized ( Figure 4B ). Apex of posterior respiratory process is differentiated in four emarginations, which persist in the 3 ^rd^ instar. In the 3 ^rd^ instar, the cuticle is strongly sclerotized, having numerous wart-like processes of different sizes surrounded by line arrangements of granulation ( Figure 4C ). In 3 ^rd^ instar larva and prepupa, the area from which each pupal spiracle emerges forms a slightly convex disc without reticulation ( Figure 4C–E ).

Pupa ( Figures 3H–I , 5A , B , D , E ). Length: 6.95–9.33 mm; maximum width: 4.05–6.16 mm ( *N* = 3). Brownish. Differing from 3 ^rd^ instar larva by being firmly attached to substrate by a pair of pupal spiracles and solid scleroti-sation on ventral side ( Figure 5A , B , D , E ). Pupal spiracles papilliform, dark brown (Figure 5A , B ), tuberculate around tips, and reticulated around basal half ( Figure 3H ). Spiracular openings simple and situated on tubercles at the tip of that structure ( Figure 3I ). Opercular opening of puparium notched dorsally ( Figure 5C ).

**Figure 6.**
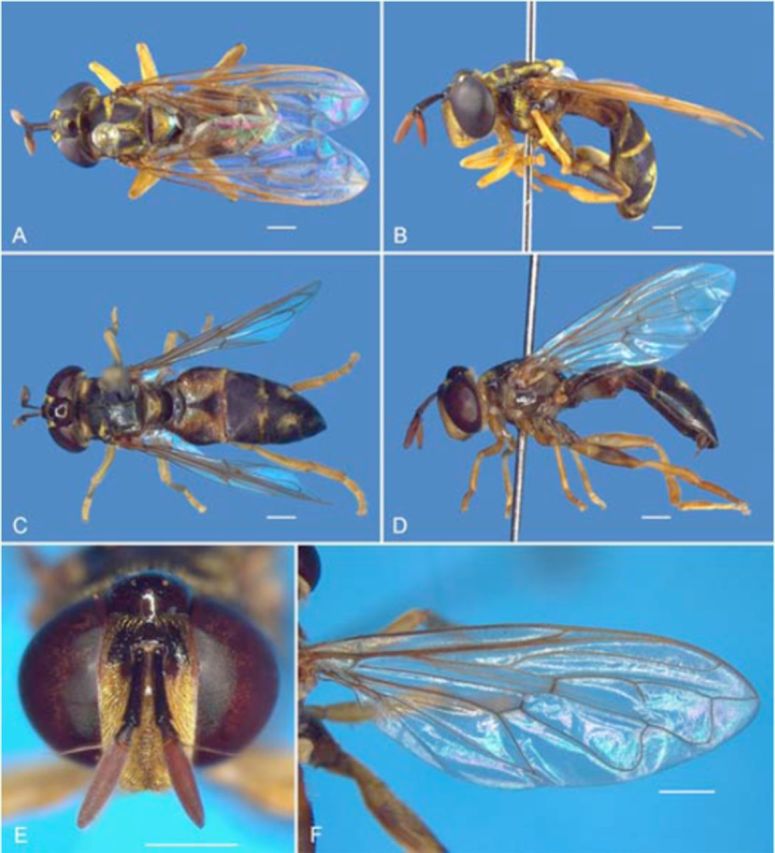
*Pseudomicrodon biluminiferus* , adult. **A.** Dorsal view, male. **B.** Lateral view, male. **C.** Dorsal view, female. **D.** Lateral view, female. **E.** Head, anterior view, female. **F.** Wing, dorsal view, male. Scale bar: 1 mm. High quality figures are available online.

**Figure 7.**
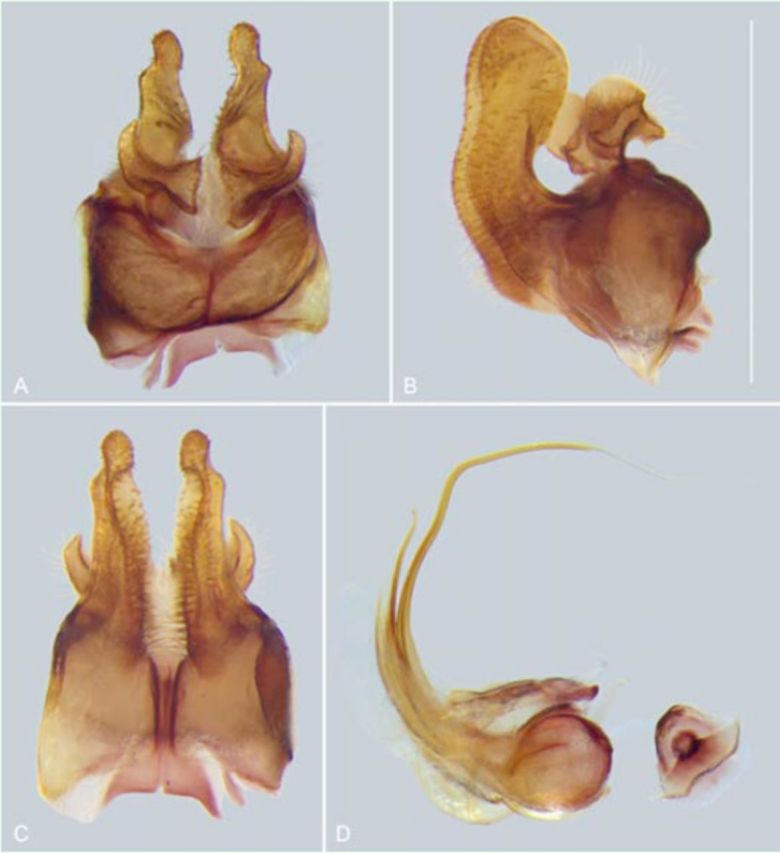
*Pseudomicrodon biluminiferus* , male genitalia. *Epandrium, surstylus,* and *circus:***A.** Ventral view. **B.** Lateral view. **C.** Dorsal view. **D.** Hypandrium and aedeagus, lateral view. Scale bar: 1 mm. High quality figures are available online.

Adult ( Figures 6A–F , 7A–D ). The holotype male from Espírito Santo state, Brazil, was deposited in Naturhistorisches Museum Wien. Hull (1944) described the specimen well, but omitted descriptions and illustrations of the male genitalia. The sexes are similar; both are dichoptic ( Figure 6 A–F), and they differ only in their genital abdominal segments. Male genitalia ( *N* = 5): hypandrium membranous; aedeagus elongated and thin, basally globose ( Figure 7D ); surstylus longer than wide, concave on internal surface ( Figure 7A , C ), arcuate in lateral view ( Figure 7B ); cercus wider than long, arcuate in dorsal view ( Figure 7A–C ).

Material examined. Three 1 ^st^ instar larvae, two 2 ^nd^ instar larvae, two 3 ^rd^ instar larvae, one prepupa, three pupae, and five puparia. Adults: eight females and five males from Santo Antônio de Lisboa, municipality of Florianópolis, Santa Catarina, Brazil.

### Host identity and infestation rate

All *P. biluminiferus* larvae, pupae, and adults were obtained from nests of ants belonging to the genus *Crematogaster* . All collected ants were identified as *C. limata* ( *N* colonies = 13). Their nests were usually found in bromeliad rosettes with broad, short, erect leaves ( Figure 1A ) that formed cavities between one another ( Figure 1B , D ). Frequently, carton-like sheaths closed the rosettes’ upper openings (remains can be seen in Video 2 at the left edge of the video frame). Sometimes, rosettes with slender, far-projecting leaves also formed such cavities at their bases, making those plants suitable nest sites, too.

Twelve of 36 systematically examined *Crematogaster* nests contained brood of *P. biluminiferus* . Twenty-four colonies of other ant genera were found in bromeliad rosettes: 2x *Brachymyrmex* , 2x *Camponotus* , 1x *Pachycondyla* , 3x *Pheidole* , 1x probably *Azteca* ; remaining records not identified because no samples were collected. Two rosettes contained signs of microdontine infestation: one *Brachymyrmex coactus* Mayr (Hymenoptera: Formicidae) nest with remains of three microdontine puparia, and Microdontinae sp. 1, as reported above. Almost all ant nests were found in rosettes of *A. nudicaulis* and *A. lindenii* . Only two *C. limata* nests (1–2 m distant from each other, therefore probably the same colony) with *Pseudomicrodon* pupae and larvae were detected in rosettes and dead infructescence stems of the bromeliad *Vriesea friburgensis* Mez var. *paludosa* (L. B. Smith) L. B. Smith (Poales: Bromeliaceae). Five infested ant nests were searched thoroughly for microdontine brood and were found to contain 2 (5) (numbers in parentheses include puparia), 2 (2), 0 (4), 13 (13), and 5 (5) brood items, with a mean of 4.4 (5.8).

### Behavioral interactions

In the laboratory colonies, *P. biluminiferus* larvae and pupae were almost completely ignored by the ants ( Videos 1 , 4–6 ). Ants were rarely observed inspecting the *Pseudomicrodon* larvae with their mandibles and antennae, and they never behaved aggressively. At times, the ants placed their own brood upon the *Pseudomicrodon* larvae and pupae or walked over them as if they were normal nest ground. When transferred to another ant nest, *Pseudomicrodon* larvae were not attacked by resident ants, whereas foreign (but conspecific) worker ants were attacked ( Video 6 , Figure 1M ). The single *Pseudomicrodon* imago that was observed upon emergence was not clearly attacked by ants while it left the nest. Upon emergence of the fly’s head, two workers pointed their abdomens toward it, probably depositing poison or alarm pheromone. Even when ants were walked over by the fly, they showed at most undirected alarming behavior by raising their gasters ( Video 1 ). Twice a 3 ^rd^ instar *Pseudomicrodon* larva was observed drawing an ant larva beneath its body ( Videos 4 , 5 ). Aside from this, no interactions between *Pseudomicrodon* brood and ants were observed.

**Videos 4 and 5.** *Pseudomicrodon biluminiferus* 3rd instar larva within alaboratory ant nest ( *Crematogaster limata* ), drawing an ant larva beneathits body. Available online at www.insectscience.org/14.38/Schmidvid4.avi and www.insectscience.org/14.38/Schmidvid5.avi

**Video 6.** Two *Pseudomicrodon biluminiferus* 2nd instar larvae transferred from one laboratory ant nest ( *Crematogaster limata* ) into another. The larva already present at the beginning of the video was placed into the nest just before the start of recording. The ants inspect the larvae but, with few exceptions, do not behave aggressively. Available onine at www.insectscience.org/14.38/Schmidvid6.avi

## Discussion

At present, the current study is the only study on preadult life history and host associations of Neotropical microdontine species, aside from that of Borgmeier (1923) , Forti et al. (2007) , and a few mere host records where the microdontine and/or ant species mostly remained unidentified. We provide new data on morphology, development, and ecology of the myrmecophilous syrphid *P. biluminiferus* that supplement the original description ( Hull 1944 ), which only gives morphological details of an adult male for one locality. To the authors’ knowledge, the current study is the second record of microdontine-ant associations involving bromeliads (after Pérez-Lachaud et al. 2014 ), and the first of microdontine larvae developing in nests of the ant species *Camponotus melanoticus* and *Crematogaster limata* .

### Biology of *Pseudomicrodon biluminiferus*

Because only relatively large 1 ^st^ instar larvae were found, freshly-hatched ones were probably missed, which might have resulted in an underestimation of the period of larval occurrence throughout the year. Unfortunately, the small data set did not allow for estimation of the duration of each larval stage.

Holarctic microdontine species usually have only one generation per year, i.e., a univoltine life cycle ( Akre et al. 1988 ). There is at least one exception ( *M. fuscipennis* ) with two or more generations ( Duffield 1981 ), and several species have been assumed to also have two or more generations ( Duffield and Thompson 1981 ). The lack of *P. biluminiferus* larvae found from mid-January to the end of February suggests either that the occasional searches during that period were not sufficient or that the adults made a pause in reproduction. If the latter is true, the life cycle of *P. biluminiferus* must be at least bivoltine. The number of generations of *P. biluminiferus* that develop per year cannot be decided without additional field data. It is unknown which developmental stages are present during the winter in southern Brazil; the lack of larvae and pupae within ant nests in the months before November suggests that it is the adults (or, less likely, eggs or 1 ^st^ instar larvae) rather than large larvae as in Holarctic species (Andries 1912; Garnett et al. 1985 ; Akre et al. 1990 ). In *M. tigrinus* , the only other South American microdontine species whose biology has been studied, adults were reported to be present in the winter months May and/or June, and larvae and pupae were found during the whole year in São Paulo State, southeastern Brazil ( Forti et al. 2007 ). This suggests that in tropical and subtropical regions, adults and/or larvae of Microdontinae might be the developmental stages that outlast climatically unfavorable times.

The observations concerning pupation and emergence concur with observations of North American and European microdontine species ( Wheeler 1908 ; Andries 1912 ; van Pelt and van Pelt 1972 ; Akre et al. 1973 , 1988; Duffield 1981; Garnett et al. 1985 ). Established observations of the number of brood items per nest ( Duffield 1981 ; Schönrogge et al. 2002 ) and of the sex ratio being near 1:1 ( Akre et al. 1973 , 1988; Duffield 1981 ) are also reinforced by the current study. The orientation of pupae toward the nest entrance (mostly in the direction of the rosette leaf tips in the field) might be an adaptation to ease leaving the nest and reaching an elevated place for wing extension. The same seems to be the case in Microdontinae sp. 1 ( Figure 1J ). In microdontine species whose adults are attacked by the host ants ( Andries 1912 ; Akre et al. 1973 ), this trait probably serves mainly to minimize contact with the ants.

Females of *P. biluminiferus* might search specifically for ant nests in bromeliad rosettes to oviposit. Herbivorous insects have long been known to use visual stimuli in host plant detection ( Prokopy and Owens 1983 ; Bernays and Chapman 1994 ), and it is well established that parasitoids may be attracted by their hosts’ preferred microhabitats even in absence of their hosts ( Godfray 1994 ). In this sense, bromeliads can be considered microhabitats with high probability of microdontine hosts occurring. Assuming that bromeliads (especially their visually conspicuous inflorescences, see below) can be recognized by the syrphid’s eyes more easily than other nest sites (e.g. dead sticks, soil, or inconspicuous cavities within other plants), searching for these plant structures could be regarded as a highly effective strategy. Nevertheless, the flies might have to apply further mechanisms to find suitable host nests within large groups of bromeliad clones, e.g. using chemical host recognition cues, as reported for the hoverfly *Microdon mutabilis* ( Schönrogge et al. 2008 ).

### Ramifications of new taxonomic placement of *Pseudomicrodon biluminiferus*

Recently, the species *Microdon biluminiferus* was transferred to the genus *Pseudomicrodon* ( Reemer and Ståhls 2013 ). Due to this new taxonomic placement of *P. biluminiferus* , the present study constitutes another novelty. All previous records of microdontine larvae preying on immature stages of their host ants apply to species of the genera *Microdon* s.s. and *Omegasyrphus* ( Reemer 2013a ). *Pseudomicrodon* is the third known genus with occurrence of ant brood predation.

### Identity and occurrence of host ants

Although not all *Crematogaster* nests containing *Pseudomicrodon* brood were identified at species level, all are considered to belong to *C. limata* . This is because no other *Crematogaster* species was found inhabiting bromeliad rosettes throughout the duration of the study, and because *C. limata* appears to be one of the most common ground-dwelling ant species at the study site ( Rosumek et al. 2008 , referred to as *Crematogaster* sp. 1), frequently visiting inflorescences of terrestrial bromeliads ( Schmid et al. 2010 ).

The mere presence of microdontine brood in an ant colony does not necessarily imply that those ants are natural hosts, because ant colonies may abandon their nests (leaving microdontine brood behind), which can then be recolonized by other species ( Wheeler 1906 , 1910; Akre et al. 1990 ; Schönrogge et al. 2000 , 2002). This possibility for recolonization renders many records based on single or rare findings of microdontine hosts doubtful. The current study shows that *C. limata* is indeed a valid host record for *P. biluminiferus* because: (1) this syrphid was repeatedly found in nests of *C. limata* , (2) no aggression on behalf of the ants toward the *Pseudomicrodon* larvae was observed, and (3) observations strongly indicate that the *Pseudomicrodon* larvae are predatory myrmecophiles that feed on brood of *C. limata* .

Longino’s (2003b) statement that large colonies of *C. limata* may be distributed over several small cavities within a small area con-curs with our observation that frequently two to four adjacent rosettes within a group of bromeliads were occupied by *C. limata* . Queens were never found (except in one small founding colony) in the present study, so there might indeed be only one ant colony scattered over several rosettes. This should be favorable to the *Pseudomicrodon* females in case they try to relocate their maternal host colony for ovipositing, as females of *M. mutabilis* ( Elmes et al. 1999 ) are known to do.

The association between *P. biluminiferus* and *C. limata* described here is not restricted to the bromeliad genus *Aechmea* . Considering the high density of bromeliad rosettes on the forest floor (up to 200 plants/ha, Müller and Marcondes 2006 ) and in the canopy, Santa Catarina Island presumably houses a large host population for local *P. biluminiferus* flies. This assumption is strengthened by the unpublished finding that 11% of *Aechmea* rosettes contained ant nests, of which 63% belonged to the genus *Crematogaster* .

Few microdontine hosts have been identified in Neotropical regions so far, and only four of them belong to the genus *Crematogaster* ( Wheeler 1924 ; Mann 1928 ; Longino 2003a ; present study). Regarding the high number of microdontine species in the Neotropics (202; Reemer and Ståhls 2013 ; Reemer 2013b ), there are certainly many more microdontine-ant associations waiting to be discovered, probably many involving *Crematogaster* (100 Neotropical species; Fernández and Sendoya 2004 ).

### Host-parasite relationship

The lack of *P. biluminiferus* brood in nests of other ant species suggests that the myrmecophile is specifically adapted to *C. limata* . The exception, remains of puparia in a *Brachymyrmex* nest, may be explained by colony turnover as described above. Desertion of *Pseudomicrodon* larvae by a previously-disturbed *Crematogaster* colony was once recorded in the field, which supports the assumption of colony turnover.

The indifferent behavior of *C. limata* ants toward the *Pseudomicrodon* larvae, together with the weak aggression toward *Pseudomicrodon* larvae from another ant nest, indicates that the *Pseudomicrodon* brood is well-adapted to live with this ant species. However, preliminary examinations of non-polar cuticular substances of *P. biluminiferus* larvae and their hosts suggested that there is no mimicry or camouflage of the ants’ chemical profiles by the parasites (unpublished data), contrary to what was reported for two North American microdontine species ( Garnett et al. 1985 ; Howard et al. 1990a , b; Stanley-Samuelson et al. 1990 ). Instead, *Pseudomicrodon* larvae, pupae, and newly-emerged adults might employ a strategy called “chemical insignificance” ( Lenoir et al. 2001 , 2012), meaning that the animals are not detectable as aliens due to the absence (or very low amounts) of “suspicious” substances. If this is true, chemical recognition of *P. biluminiferus* by the ants might not have been the selective force that drove this syrphid into specialization with *C. limata* as host. Instead, other elements might have been involved, e.g. host localization. However, the cuticular chemistry of this *Pseudomicrodon* species must be analyzed more thoroughly before confident conclusions can be drawn.

Garnett et al. (1985) reported 1 ^st^ and 2 ^nd^ instar larvae of North American microdontine species being carried by host ants when disturbed and described a specific “cocoon mimicry” actively performed by microdontine larvae, revealing a behavioral adaptation of the parasites to their hosts. There were no observations of ants carrying *Pseudomicrodon* brood in the present study, even though colonies containing those parasites were frequently disturbed in the field. The large 3 ^rd^ instar was mainly recognized, and occasionally 2 ^nd^ instar larvae. The fact that small *Pseudomicrodon* larvae were never found might be explained not only by absence of these stages but alternatively by the existence of similar brood mimicry, causing 1 ^st^ instar larvae to be overlooked upon inspection of ant colonies. The former case seems to be more likely because numbers of *Pseudomicrodon* larvae did not increase in colonies kept in the laboratory for weeks. Either way, occurrence and behavior of the small stages of *P. biluminiferus* deserve closer examination.

The single observation of a newly-emerged adult leaving a laboratory nest undisturbed by the surrounding ants corresponds to a similar case described by Wheeler (1908) . However, Andries (1912) and Akre et al. (1973) observed that microdontine adults were killed immediately after emergence, and Wheeler (1908) reported that they were attacked later during expansion of their wings. Whether this is the case in *P. biluminiferus* could not be determined because no contact between adults and host ants was observed outside the nests.

Our observations corroborate several reports of microdontine larvae feeding on their hosts’ brood (e.g., van Pelt and van Pelt 1972 ; Duffield 1981; Garnett et al. 1985 ; Barr 1995 ; Pérez-Lachaud et al. 2014 ). Nevertheless, this is not necessarily true for all microdontine species that have also been observed or assumed to feed on coccids ( Borgmeier 1923 , 1953), infrabuccal pellets of ants ( Wheeler 1908 ; Donisthorpe 1927 ), fungi and tree sap ( Garnett et al. 1985 ), or detritus ( Forti et al. 2007 ). The infestation rate of *C. limata* colonies by *P. biluminiferus* (33%) lies well within those reported for Holarctic microdontine species, which are 33–50% ( van Pelt and van Pelt 1972 ) and 16% ( Akre et al. 1973 ).

### Single discovery of Microdontinae sp. 1

Throughout the study period, Microdontinae sp. 1 was recorded only once. The same might be true for its putative host species, *Camponotus melanoticus* , although not all ants found in bromeliads were identified. Concordantly, in a prior extensive census of ants within bromeliads in the study area, *C. melanoticus* had been found only once in one plant of *A. nudicaulis* ( Rosumek et al. 2008 ; A. Zillikens, J. Steiner, unpublished data). This implies either that the host and probably also the parasite occur only rarely in the study area, or that *C. melanoticus* colonies infrequently live in bromeliads. The discovery of *C. melanoticus* and *Crematogaster limata* nesting in the same bromeliad rosette, infested by different microdontine species, suggests species specificity of the microdontine-ant associations. Furthermore, it might be a case of facultative parabiosis (Weber 1943), in which two ant species coexist without being dependent on each other. Hopefully, in future studies, adults of Microdontinae sp. 1 will be reared for identifying the species and exploring this unknown microdontine-ant relationship further.

### The role of the bromeliads

Colonies of *C. limata* have been reported as not showing specific preferences for nesting sites ( Longino 2003b ). So, as long as thorough examinations do not reveal the opposite for the local population on Santa Catarina Island, it must be assumed that these ants occur frequently outside of bromeliads, providing even more potential host nests for *P. biluminiferus* than the current study indicates. Depending on cues the adult flies use for host location (Godfray 1994), ant nests in bromeliads might be easier to detect from a distance because of visual stimuli provided by the rosettes, frequently enhanced by the conspicuously-colored inflorescences ( Figure 1A–C ) that appear during the syrphid’s reproductive season ( Schmid et al. 2010 ; Dorneles et al. 2011 ). Moreover, the vase-like shape of the bromeliad rosettes might concentrate olfactory signals emitted by the host colonies between the leaves, thereby impeding long-range diffusion of chemical cues while simultaneously facilitating short-range host recognition (see Schönrogge et al. 2008 for use of chemical signals by *M. mutabilis* ). The possibility of bromeliads playing a major role in this microdontine-ant association surely warrants further investigation.

### Conclusions

The biology of *P. b iluminiferus* is largely similar to that of species in Holarctic regions, from general biology to predatory behavior and host specificity. However, two traits of this syrphid may differ from established knowledge: the overwintering developmental stage (presumably adults) and the (probably specific) association with bromeliads. The latter, together with the discovery of Microdontinae sp. 1, adds two more cases to the small list of microdontine species associated with ants that reliably nest in certain types of plants ( Wheeler 1924 ; Hocking 1970 ). Due to the high number of known microdontine species, especially from the Neotropics, and the low number of studies on microdontine-ant associations in this region, there is a high potential for discovery of other relationships (e.g., with parasitoids of Microdontinae, as reported recently by Hansson et al. 2011 ). This indicates that the ecologies of the inconspicuous microdontine flies and the well-studied ant family Formicidae have not been sufficiently investigated to obtain a deep understanding of this interesting host-parasite system. We hope that the data provided in the current study will encourage future research in this area.

## References

[R1] Akre RD Alpert G Alpert T . 1973 . Life cycle and behavior of *Microdon cothurnatus* in Washington (Diptera: Syrphidae) . Journal of the Kansas Entomological Society 46 ( 3 ): 327 – 338 .

[R2] Akre RD Garnett WB Zack RS . 1988 . Biology and behavior of *Microdon piperi* in the Pacific Northwest (Diptera: Syrphidae) . Journal of the Kansas Entomological Society 61 ( 4 ): 441 – 452 .

[R3] Akre RD Garnett WB Zack RS . 1990 . Ant hosts of *Microdon* (Diptera: Syrphidae) in the Pacific Northwest . Journal of the Kansas Entomological Society 63 ( 1 ): 175 – 178 .

[R4] Andries M . 1912 . Zur Systematik, Biologie und Entwicklung von *Microdon* Meigen . Zeitschrift für Wissenschaftliche Zoologie 103 : 300 – 361 .

[R5] Barr B . 1995 . Feeding behaviour and mouthpart structure of larvae of *Microdon eggeri* and *Microdon mutabilis* (Diptera, Syrphidae) . Dipterists Digest 2 : 31 – 36 .

[R6] Benzing DH . 2000 . Bromeliaceae – Profile of an adaptive radiation . Cambridge University Press .

[R7] Bernays EA Chapman RF . 1994 . Host-Plant Selection by Phytophagous Insects . Chapman & Hall .

[R8] Borgmeier T . 1923 . Beitrag zur Biologie der Feuerameise und ihrer Gäste ( *Solenopsis geminata saevissima* Sm.) . Zeitschrift des Deutschen Vereins fur Wissenschaft und Kunst São Paulo 3 : 1 – 9 .

[R9] Borgmeier T . 1953 . Syrphidenlarven in Ameisennestern . Naturwissenschaften 40 ( 2 ): 36 .

[R10] Buschinger A . 1998 . Als Wolf im Schafspelz enttarnt – Die Larven der Schwebfliege *Microdon* . Ameisenschutz aktuell 12 ( 2 ): 47 – 51 .

[R11] Camargo RX Oliveira PS . 2012 . Natural history of the Neotropical arboreal ant, *Odontomachus hastatus* : Nest sites, foraging schedule, and diet . Journal of Insect Science 12 : 48 . Available online: www.insectscience.org/12.48/22957686 10.1673/031.012.4801 PMC3476954

[R12] Cheng X-Y Thompson FC. 2008 . A generic conspectus of the Microdontinae (Diptera: Syrphidae) with the description of two new genera from Africa and China . Zootaxa 1879 : 21 – 48 .

[R13] Dejean A Olmsted I . 1997 . Ecological studies on *Aechmea bracteata* (Swartz) (Bromeliaceae) . Journal of Natural History 31 : 1313 – 1334 .

[R14] Dixon TJ . 1960 . Key to descriptions of third instar larvae of some Syrphidae (Diptera) occurring in Britain . Transactions of the Royal Entomological Society of London 112 : 345 – 379 .

[R15] Donisthorpe H . 1927 . The guests of British ants – their habits and life-histories . George Routledge and Sons .

[R16] Dorneles LL Zillikens A Harter-Marques B Steiner J . 2011 . Effective pollinators among the diverse flower visitors of the bromeliad *Aechmea lindenii* in south Brazilian Atlantic rain forests . Entomologia Generalis 33 ( 3 ): 149 – 164 .

[R17] Duffield RM . 1981 . Biology of *Microdon fuscipennis* (Diptera: Syrphidae) with interpretations of the reproductive strategies of *Microdon* species found north of Mexico . Proceedings of the Entomological Society of Washington 83 ( 4 ): 716 – 724 .

[R18] Duffield RM Thompson FC . 1981 . Behavioral strategies and ant associations of the *Microdon* species found north of Mexico. Tables 2 & 3 in Duffield, R. M. 1981. Biology of *Microdon fuscipennis* (Diptera: Syrphidae) with interpretations of the reproductive strategies of *Microdon* species found north of Mexico . Proceedings of the Entomological Society of Washington 83 ( 4 ): 716 – 724 .

[R19] Elmes GW Barr B Thomas JA Clarke RT . 1999 . Extreme host specificity by *Microdon mutabilis* (Diptera: Syrphidae), a social parasite of ants . Proceedings of the Royal Society of London B Biological Sciences 266 : 447 – 453 .

[R20] Fernández F Sendoya S. 2004 . List of Neotropical ants (Hymenoptera: Formicidae) . Biota Colombiana 5 ( 1 ): 3 – 93 .

[R21] Forti LC Camargo RS Verza SS Andrade APP Fujihara RT Lopes JFS . 2007 . *Microdon tigrinus* Curran, 1940 (Diptera, Syrphidae): Populational Fluctuation and Specificity to the Nest of *Acromyrmex coronatus* (Hymenoptera: Formicidae) . Sociobiology 50 : 1 – 11 .

[R22] Frank JH Lounibos LP . 2008 . Insects and allies associated with bromeliads: a review . Terrestrial Arthropod Reviews 1 : 125 – 153 . 10.1163/187498308X414742 PMC2832612 20209047

[R23] Garnett WB Akre RD Sehlke G . 1985 . Cocoon mimicry and predation by myrmecophilous Diptera (Diptera: Syrphidae) . Florida Entomologist 68 ( 4 ): 615 – 621 .

[R24] Garnett WB Akre RD Zack RS . 1990 . External morphology of four species of *Microdon* immatures (Diptera: Syrphidae) from the Pacific Northwest . Annals of the Entomological Society of America 83 : 68 – 80 .

[R25] Godfray HCJ . 1994 . Parasitoids: Behavioral and Evolutionary Ecology . Princeton University Press .

[R26] Greene CT . 1923a . A contribution to the biology of North American Diptera . Proceedings of the Entomological Society of Washington 25 : 82 – 91 .

[R27] Greene CT . 1923b . The larva and pupa of *Microdon megalogaster* Snow . Proceedings of the Entomological Society of Washington 25 : 140 – 141 .

[R28] Greene CT . 1955 . Larvae and pupae of the genera *Microdon* and *Mixogaster* (Diptera: Syrphidae) . Transactions of the American Entomological Society 81 : 1 – 20 .

[R29] Hansson C Lachaud J-P Pérez-Lachaud G. 2011 . Entedoninae wasps (Hymenoptera, Chalcidoidea, Eulophidae) associated with ants (Hymenoptera, Formicidae) in tropical America, with new species and notes on their biology . ZooKeys 134 : 65 – 82 . DOI: 10.3897/zookeys.134.1653 10.3897/zookeys.134.1653 PMC3229211 22140342

[R30] Hironaga T Maruyama M . 2004 . The myrmecophilous hoverfly genus *Microdon* (Diptera, Syrphidae, Microdontinae) in Hokkaidô, Japan, with descriptions of four new species . Bulletin of the National Science Museum Series A 30 ( 2 ): 87 – 103 .

[R31] Hocking B . 1970 . Insect associations with the swollen thorn acacias . Transactions of the Royal Entomological Society of London 122 ( 7 ): 211 – 255 .

[R32] Hölldobler B Wilson EO. 1990 . The Ants . Harvard University Press .

[R33] Hölldobler B Wilson EO. 2009 . The superorganism: The Beauty, Elegance, and Strangeness of Insect Societies . W.W. Norton .

[R34] Howard RW Akre RD Garnett WB . 1990a . Chemical mimicry of an obligate predator of carpenter ants (Hymenoptera: Formicidae) . Annals of the Entomological Society of America 83 : 607 – 16 .

[R35] Howard RW Stanley-Samuelson OW Akre RD . 1990b . Biosynthesis and chemical mimicry of cuticular hydrocarbons from an obligate predator, *Microdon albicomatus* Novak (Diptera: Syrphidae) and its ant prey, *Myrmica incompleta* Provancher (Hymenoptera: Formicidae) . Journal of the Kansas Entomological Society 63 : 437 – 43 .

[R36] Hull FM . 1944 . Studies on flower flies (Syrphidae) in the Vienna Museum of Natural History . Journal of the Washington Academy of Sciences 34 : 398 – 404 .

[R37] Jordan KHC . 1968 . Biologische Beobachtungen an *Microdon* (Diptera: Syrphidae) . Entomologische Berichte (Berlin) 12 ( 1 ): 15 – 18 .

[R38] Kistner DH . 1982 . The social insects’ bestiary. In: Hermann HR, Editor . Social insects, volume 3 . pp. 1 – 244 . Academic Press.

[R39] Lachaud J-P Lenoir A Witte V. 2012 . Ants and Their Parasites . Psyche 2012 : Article ID 342157. DOI: 10.1155/2012/342157

[R40] Lenoir A D’Ettorre P Errard C Hefetz A. 2001 . Chemical ecology and social parasitism in ants . Annual Review of Entomology 46 : 573 – 599 . 10.1146/annurev.ento.46.1.573 11112180

[R41] Lenoir A Chalon Q Carvajal A Ruel C Barroso Á Lackner T Boulay R. 2012 . Chemical integration of myrmecophilous guests in *Aphaenogaster* ant nests . Psyche 2012 : Article ID 840860. DOI: 10.1155/2012/840860

[R42] Longino JT . 2003a . Formicidae: *Crematogaster brasiliensis* . Available online: http://academic.evergreen.edu/projects/ants/GENERA/crematogaster/species/brasiliensis/brasiliensis.html

[R43] Longino JT . 2003b . Formicidae: *Crematogaster limata* . Available online: http://academic.evergreen.edu/projects/ants/GENERA/crematogaster/species/limata/limata.html

[R44] Luederwaldt H. 1926 . Observações biológicas sobre as formigas brasileiras especialmente do Estado de São Paulo . Revista do Museu Paulista 14 : 185 – 304 + 5 plates.

[R45] Mann WM . 1928 . A new *Microdon* from Panama . Psyche 35 ( 3 ): 168 – 170 . DOI: 10.1155/1928/73806

[R46] Müller GA Marcondes CB. 2006 . Bromeliad-associated mosquitoes from Atlantic forest in Santa Catarina Island, southern Brazil (Diptera, Culicidae), with new records for the State of Santa Catarina . Iheringia, Série Zoologia 96 : 315 – 319 .

[R47] Novak JA Akre RD Garnett WB . 1977 . Keys to adults and puparia of five species of *Microdon* (Diptera: Syrphidae) from eastern Washington and northern Idaho, with descriptions of a new species . Canadian Entomologist 109 : 663 – 668 .

[R48] Pérez-Lachaud G Jervis MA Reemer M Lachaud J-P. 2014 . An unusual, but not unexpected, evolutionary step taken by syrphid flies: the first record of true primary parasitoidism of ants by Microdontinae . Biological Journal of the Linnean Society 111 : 462 – 472 . DOI: 10.1111/bij.12220.

[R49] Prokopy RJ Owens ED . 1983 . Visual detection of plants by herbivorous insects . Annual Review of Entomology 28 : 337 – 364 .

[R50] Reemer M . 2013a . Review and phylogenetic evaluation of associations between Microdontinae (Diptera: Syrphidae) and ants (Hymenoptera: Formicidae) . Psyche 2013, Article ID 538316. DOI: 10.1155/2013/538316

[R51] Reemer M . 2013b . Taxonomic exploration of Neotropical Microdontinae (Diptera: Syrphidae) mimicking stingless bees . Zootaxa 3697 ( 1 ): 1 – 88 . DOI: 10.11646/zootaxa.3697.1.1 26079022 10.11646/zootaxa.3697.1.1

[R52] Reemer M Ståhls G. 2013 . Generic revision and species classification of the Microdontinae (Diptera, Syrphidae) . ZooKeys 288 : 1 – 213 . DOI: 10.3897/zookeys.288.4095 10.3897/zookeys.288.4095 PMC3690914 23798897

[R53] Roberts MJ . 1970 . The structure of the mouthparts of syrphid larvae (Diptera) in relation to feeding habits . Acta Zoologica 51 : 43 – 65 .

[R54] Rosumek FB Ulysséa MA Lopes BC Steiner J Zillikens A. 2008 . Formigas de solo e de bromélias em uma área de Mata Atlântica, Ilha de Santa Catarina, sul do Brasil: Levantamento de espécies e novos registros . Biotemas 21 : 81 – 89 .

[R55] Rotheray GE Gilbert FS . 1999 . Phylogeny of Palaearctic Syrphidae (Diptera): evidence from larval stages . Zoological Journal of the Linnean Society 127 : 1 – 112 .

[R56] Schmid VS Schmid S Steiner J Zillikens A . 2010 . High diversity of ants foraging on extrafloral nectar of bromeliads in the Atlantic rainforest of southern Brazil . Studies on Neotropical Fauna and Environment 45 : 39 – 54 .

[R57] Schönrogge K Wardlaw JC Thomas JA Elmes GW. 2000 . Polymorphic growth rates in myrmecophilous insects . Proceedings of the Royal Society of London B Biological Sciences 267 : 771 – 777 . 10.1098/rspb.2000.1070 PMC1690603 10819146

[R58] Schönrogge K Barr B Wardlaw JC Napper EKV Gardner MG Breen J Elmes GW Thomas JA. 2002 . When rare species become endangered: cryptic speciation in myrmecophilous hoverflies . Biological Journal of the Linnean Society 75 : 291 – 300 .

[R59] Schönrogge K Napper EKV Birkett MA Woodcock CM Pickett JA Wadhams LJ Thomas JA. 2008 . Host recognition by the specialist hoverfly *Microdon mutabilis* , a social parasite of the ant *Formica lemani* . Journal of Chemical Ecology 34 : 168 – 178 . 18185959 10.1007/s10886-007-9417-8

[R60] Sharp D . 1899 . Insects, part II. In: Harmer SF, Shipley AE, Editors . Cambridge Natural History Vol. VI. Macmillan and Co. Available online: http://archive.org/download/cambridgenatural06harm/cambridgenatural06harm.pdf

[R61] Speiser P . 1913 . Über einige Syrphiden und zwei für die Deutsche Fauna neue Clythiiden . Jahrbücher des Nassauischen Vereins für Naturkunde 66 : 117 – 146 .

[R62] Stanley-Samuelson DW Howard RW Akre RD . 1990 . Nutritional interactions revealed by tissue fatty acid profiles of an obligate myrmecophilus predator, *Microdon albicomatus* , and its prey, *Myrmica incompleta* (Diptera: Syrphidae) (Hymenoptera: Formicidae) . Annals of the Entomological Society of America 83 : 1108 – 1115 .

[R63] Thompson FC . 1999 . A key to the genera of the flower flies (Diptera: Syrphidae) of the neotropical region including descriptions of new genera and species and a glossary of taxonomic terms . Contributions on Entomology International 3 : 319 – 378 .

[R64] van Pelt AF van Pelt SA. 1972 . *Microdon* (Diptera: Syrphidae) in nests of *Monomorium* (Hymenoptera: Formicidae) in Texas . Annals of the Entomological Society of America 65 ( 4 ): 977 – 979 .

[R65] Wasmann E . 1894 . Kritisches Verzeichniss der myrmekophilen und termitophilen Arthropoden. Mit Angabe der Lebensweise und mit Beschreibung neuer Arten. Verlag von Felix L. Dames.

[R66] Weber NA . 1943 . Parabiosis in Neotropical "Ant Gardens" . Ecology 24 ( 3 ): 400 – 404 .

[R67] Wheeler WM . 1906 . On the founding of colonies by queen ants, with special reference to the parasitic and slave-making species . Bulletin of the American Museum of Natural History 22 : 33 – 105 .

[R68] Wheeler WM . 1908 . Studies of Myrmecophiles III. *Microdon* . Journal of the New York Entomological Society 16 : 202 – 213 .

[R69] Wheeler WM . 1910 . Ants: Their Structure, Development, and Behavior . Columbia University Press .

[R70] Wheeler WM . 1924 . Two extraordinary larval myrmecophiles from Panama . Proceedings of the National Academy of Sciences 10 ( 6 ): 237 – 244 . 10.1073/pnas.10.6.237 PMC1085630 16586933

[R71] Wilson EO . 1971 . The insect societies . Belknap Press .

